# Gut Microbiota Altered in Mild Cognitive Impairment Compared With Normal Cognition in Sporadic Parkinson's Disease

**DOI:** 10.3389/fneur.2020.00137

**Published:** 2020-02-25

**Authors:** Tengzhu Ren, Yuyuan Gao, Yihui Qiu, Shuolin Jiang, Qingxi Zhang, Jiahui Zhang, Limin Wang, Yuhu Zhang, Lijuan Wang, Kun Nie

**Affiliations:** Department of Neurology, Neuroscience Institute, Guangdong Provincial People's Hospital, Guangdong Academy of Medical Sciences, Guangdong, China

**Keywords:** Parkinson's disease, cognition impairment, gut micro biome, short fatty acids, high throughput sequencing

## Abstract

**Background and Aim:** Gut bacteria play an important role in the pathogenesis of Parkinson's disease (PD). However, the alteration of fecal microbiota in PD with cognitive impairment remains unexplored. This study aimed to explore whether the gut microbiota of patients with PD having mild cognitive impairment (PD-MCI) were different from those with PD having normal cognition (PD-NC) and from healthy controls (HC). Also, the study probed the association between altered gut microbiota and cognitive ability in patients with PD.

**Methods:** The fecal bacteria composition and short-chain fatty acids of 13 patients with PD-MCI, 14 patients with PD-NC, and 13 healthy spouses were analyzed using 16S ribosomal RNA sequencing and gas chromatography–mass spectrometry.

**Results:** Compared with HC, the fecal microbial diversities increased in patients with PD-MCI and PD-NC. After adjusting the influence of age, sex, body mass index, education, and constipation using the statistical method, the relative abundances of two families (Rikenellaceae and Ruminococcaceae) and four genera (*Alistipes, Barnesiella, Butyricimonas*, and *Odoribacter*) were found to be higher in the feces of the PD-MCI group compared with the other two groups. Moreover, the abundance of genus *Blautia* and *Ruminococcus* decreased obviously in the PD-MCI group compared with the PD-NC group. Further, the abundance of genera *Butyricimonas, Barnesiella, Alistipes, Odoribacter*, and *Ruminococcus* negatively correlated with cognition ability.

**Conclusion:** Compared with HC and patients with PD-NC, the gut microbiota of patients with PD-MCI was significantly altered, particularly manifesting in enriched genera from Porphyromonadaceae family and decreased the abundance of genera *Blautia* and *Ruminococcus*.

## Introduction

Parkinson's disease (PD) is the most prevalent neurodegenerative motor disease. Cognitive impairment is a frequent complication of the non-motor symptoms in PD, commonly described as PD with mild cognitive impairment (PD-MCI) and PD dementia (PDD), and is recognized to worsen the outcomes. Early studies indicated dementia prevalence of 15–20% after 5 years and 46% after 10 years (1, 2). The Movement Disorder Society (MDS) Task Force concluded that PD-MCI was common in non-demented patients (mean prevalence, 27%; range, 19–38%) and associated with the subsequent development of PDD (3).

Recently, converging lines of evidence supported the hypothesis that gut microbiota were associated with the pathogenesis of PD (4, 5). Many of these studies focused on the composition of gut microbiota (6, 7) and the bacterial metabolites, short-chain fatty acids (SCFAs) (8). According to Braak's hypothesis (9), the accumulation of aberrant alpha-synuclein (α-Syn) is initiated in the gut and propagated via the vagus nerve to the brain. Recent animal studies also confirmed that gut bacteria regulated movement disorders by impacting neuroinflammation and aggregation of α-Syn, supporting Braak's hypothesis in the etiology of PD (10). Nonetheless, no previous study investigated the composition of fecal microbiota in PD-MCI. This study hypothesized that fecal microbiota of patients with PD-MCI differed from those of matched healthy controls (HC) and patients with PD having normal cognition (PD-NC).

## Methods

### Recruitment

Ethics approval and written informed consent were obtained from the hospital and the patients, respectively. The patients with PD were recruited and assessed in the Department of Neurology at the Guangdong Provincial People's Hospital, Guangdong Province, China (from June 2018 to January 2019). All patients eligible for this study were diagnosed for PD according to the UK Brain Bank criteria (11). Of these patients, 14 were clinically diagnosed with PD-NC and 13 with PD-MCI based on the MDS Task Force Guidelines (12). Moreover, 13 age-matched healthy spouses of the recruited patients were enrolled as controls (Figure S1).

### Clinical Assessment

Clinical data were collected through face-to-face interviews with movement disorder specialists. Each participant's weight and height were measured, and the body mass index (BMI) was calculated. PD clinical characteristics included disease duration, education, motor and non-motor symptoms, and medication. The part III scores of MDS-Unified Parkinson's Disease Rating Scale (MDS-UPDRS) and Hoehn and Yahr stage (H-Y stage) were analyzed during the “on” state. The PD-related non-motor symptoms were evaluated using the Parkinson's Disease Questionnaire (PDQ-39) and activities of daily living (ADL). Constipation was assessed using the Wexner constipation score. Cognition abilities were estimated using the Mini-Mental State Examination (MMSE) and Montreal Cognitive Assessment (MoCA), and the scores were obtained from two other neuropsychological tests in each of the five cognitive domains. On the day of the stool sample collection, all the participants completed a questionnaire assessing their dietary habits in the last month, including the consumption of caffeine and alcohol.

### 16S rRNA Amplicons and SCFAs Analysis

Each study participant was given a fecal collection container to collect a fecal sample. The containers were stored at −80°C until DNA extraction. The DNA was extracted using a QIAamp DNA Stool Mini Kit (Qiagen, Hilden, Germany). Polymerase chain reaction (PCR) amplification of *16S rRNA* genes was performed with general bacterial primers (515F 5′-GTGCCAGCMGCCGCGGTAA-3′and 926R 5′-CCGTCAATTCMTTTGAGTTT-3′). Prior to library pooling, the barcoded PCR products were purified using a DNA gel extraction kit (Axygen, China) and quantified using the FTC-3000 real-time PCR. The *16S rRNA* amplicon (V3–V4 regions) sequencing analysis was performed using an Illumina MiSeq 2 × 300bp (MiSeq v3 Reagent Kit, CA, USA). The 16S sequences were analyzed using a combination of mothur (version 1.33.3), UPARSE (usearch version v8.1.1756), and R software (version 3.2.3). The demultiplexed reads were clustered at 97% sequence identity into operational taxonomic units (OTUs) using the UPARSE pipeline. The OTU representative sequences were selected and their taxonomies were assigned against the Silva 128 database with a confidence score ≥0.6 using the classify.seqs command in mothur. The OTU taxonomies (from phylum to genera) were determined based on National Center for Biotechnology Information. The measurement of SCFAs was carried out using the Gas Chromatography and Mass Spectrometry (GC-MS) analysis and a single quadrupole mass spectrometer equipped with 6890N GC (Agilent Technologies, CA, USA). Seven SCFA standards were obtained from Sigma–Aldrich (MO, USA) and Sinopharm Chemical Reagent Co., Ltd (Shanghai, China) at a minimum purity of 98%. The GC was fitted with a capillary column Agilent HP-INNOWAX (30 m × 0.25 mm) (Agilent Technologies).

### Bioinformatic and Statistical Analysis

The SPSS (version 20.0, SPSS Inc., IL, USA) and R software (version 3.2.3, the R Project for Statistical Computing) were used for the statistical analysis of data. The normality test was conducted using the Shapiro–Wilk test. The three groups were compared using the one-way analysis of variance and Pearson's chi–square test for quantitative and categorical variables, respectively. Subsequently, the *post hoc* Bonferroni adjustments were applied to account for multiple comparisons, with alpha set at 0.0167. The differences between PD-NC and PD-MCI groups were compared using the Student *t*-test and Pearson's chi-square test for quantitative and categorical variables, respectively, with alpha set at 0.05. Both alpha-diversity (Chao, Shannon, Simpson, sobs indexes, and so on) and beta-diversity metrics (unweighted UniFrac ANOSIM indexes, weighted UniFrac ANOSIM indexes, and PERMANOVA analysis) were calculated using Quantitative Insights into Microbial Ecology (13, 14). Alpha-and beta-diversity analyses were performed using mothur and visualized using principal coordinate analysis. Differences in abundance (at multiple taxonomic levels) among three groups were detected using a Kruskal–Wallis test. The linear discriminant analysis (LDA) effect size method was used to characterize the taxa with statistical significance and biological relevance (13, 15). Differences in significant bacterial communities among the three groups and between the clinical parameters were evaluated using a generalized linear model (GLM) (16). An OTU normalized by DESeq table was used to infer microbiota metabolic functions using the Phylogenetic Investigation of Communities by Reconstruction of Unobserved States (PICRUSt). The OTU tables were normalized by copy number, and functions were predicted using the Kyoto Encyclopedia of Genes and Genomes (KEGG) orthologs (5). Significant KEGG pathways at level 2 and 3 for the fecal microbiome of the three groups identified by STAMP software. In STAMP, differences in abundances among the PD-MCI, PD–NC and healthy groups were compared using the Kruskal–Wallis test. The Kruskal–Wallis test was also used for testing the difference in SCFAs between the three groups. Correlations between clinical parameters as well as significant bacterial communities and SCFAs for 40 participants were calculated using Spearman's rank-correlation analysis.

## Results

### Demographics

A total of 40 Cantonese people were recruited for the study. Both the patients and the controls reported to be omnivores with a conventional diverse diet and without any dietary restrictions. No significant differences in age, BMI, education, and ADL scores were found among the three groups. Moreover, no significant discrepancy in PD disease duration, MDS-UPDRS-III score, H-Y stage, anti-Parkinson medicine intake, and PDQ-39 scores was found between the PD-NC and PD-MCI groups. Significant differences in the MMSE and MoCA scores were found among the three groups (*P* = 0.014; *P* < 0.001). Moreover, obvious differences in MoCA scores were found between the PD-MCI and HC groups (*P* < 0.001, *P*_corr_ < 0.001) as well as between the PD-MCI and PD-NC groups (*P* < 0.001, *P*_corr_ < 0.001), while no difference was found between the PD-NC and HC groups (*P* > 1.000, *P* = 0.391). The sex difference reflected higher prevalence of PD in men and greater participation of women as volunteers. Further, a higher proportion of patients reported constipation in the PD-NC and PD-MCI groups compared with the HC group. However, the influence of sex and constipation were corrected after GLM analysis (Table 1).

**Table 1:** Selected demographic and clinical parameters of HC group, PD-NC group and PD-MCI group.

		**HC (*n* = 13)**	**PD-NC (*n* = 14)**	**PD-MCI (*n* = 13)**	***P-value***	***Pcorr (Bonferroni corrected)***		
						**PD-NC VS.PD-MCI**	**PD-NC VS. HC**	**PD-MCI VS. HC**
Age ^a^		63.00 (8.76)	60.00 (9.20)	65.23 (10.96)	0.379	0.506	>1.000	>1.000
Sex^b^	F	10	4	4	0.019 (7.943)	−0.901 (0.016)^1^	−0.012 (6.312)^2^	−0.018 (5.571)^3^
	M	3	10	9				
BMI^a^		22.67 (2.06)	22.63 (2.52)	22.74 (2.62)	0.993	>1.000	>1.000	>1.000
Education^a^		10.46 (3.53)	13.93 (2.62)	9.08 (4.46)	0.046	0.048	>1.000	0.242
ADL^a^		14.00 (0.00)	16.36 (4.24)	19.77 (10.86)	0.099	0.577	>1.000	0.101
MMSE^a^		28.54 (1.56)	28.00 (1.67)	26.38 (2.40)	0.014	0.087	>1.000	0.016
MoCA^a^		27.23 (1.53)	26.07 (1.77)	20.08 (2.43)	<0.001	<0.001	0.391	<0.001
Wexner score^a^		3.92 (2.99)	8.92 (2.02)	8.46 (1.98)	<0.001	>1.000	<0.001	<0.001
Duration^c^		–	5.64 (3.34)	7.00 (8.07)	0.568 (0.579)			
H-Y stage^c^		–	1.89 (0.49)	1.80 (0.43)	0.637 (−0.478)			
MDS-UPDRS III^c^		–	30.07 (14.01)	30.08 (14.40)	0.999 (0.001)			
PDQ-39^c^		–	27.29 (19.44)	32.46 (19.16)	0.493 (0.696)			
Anti-Parkinson medicine^b^	Y	–	10	10	0.745 (0.106)			
	N	–	4	3				
COMT- inhibitors^b^	Y	–	3	2	0.686 (0.163)			
	N	–	11	11				
Alcohol^b^	Y	2	1	0	0.329 (2.222)			
	N	11	13	13				
Coffeine^b^	Y	6	4	2	0.229 (2.951)			
	N	7	10	11				

Data are shown as mean (SD).

a Means with One-way ANOVA. Pcorr denotes values corrected for multiple comparisons using the Bonferroni method. Alpha was set at 0.0167.

b Means with Pearson's Chi-square test. 1, 2, and 3 mean difference of sex between PD-MCI and PD-NC group, PD-NC and HC group as well as PD-MCI and HC group, respectively. Alpha was set at 0.05.

c *Means with student's t-test. Alpha was set at 0.05*.

### Alpha and Beta-Diversity

**Figure 1:**
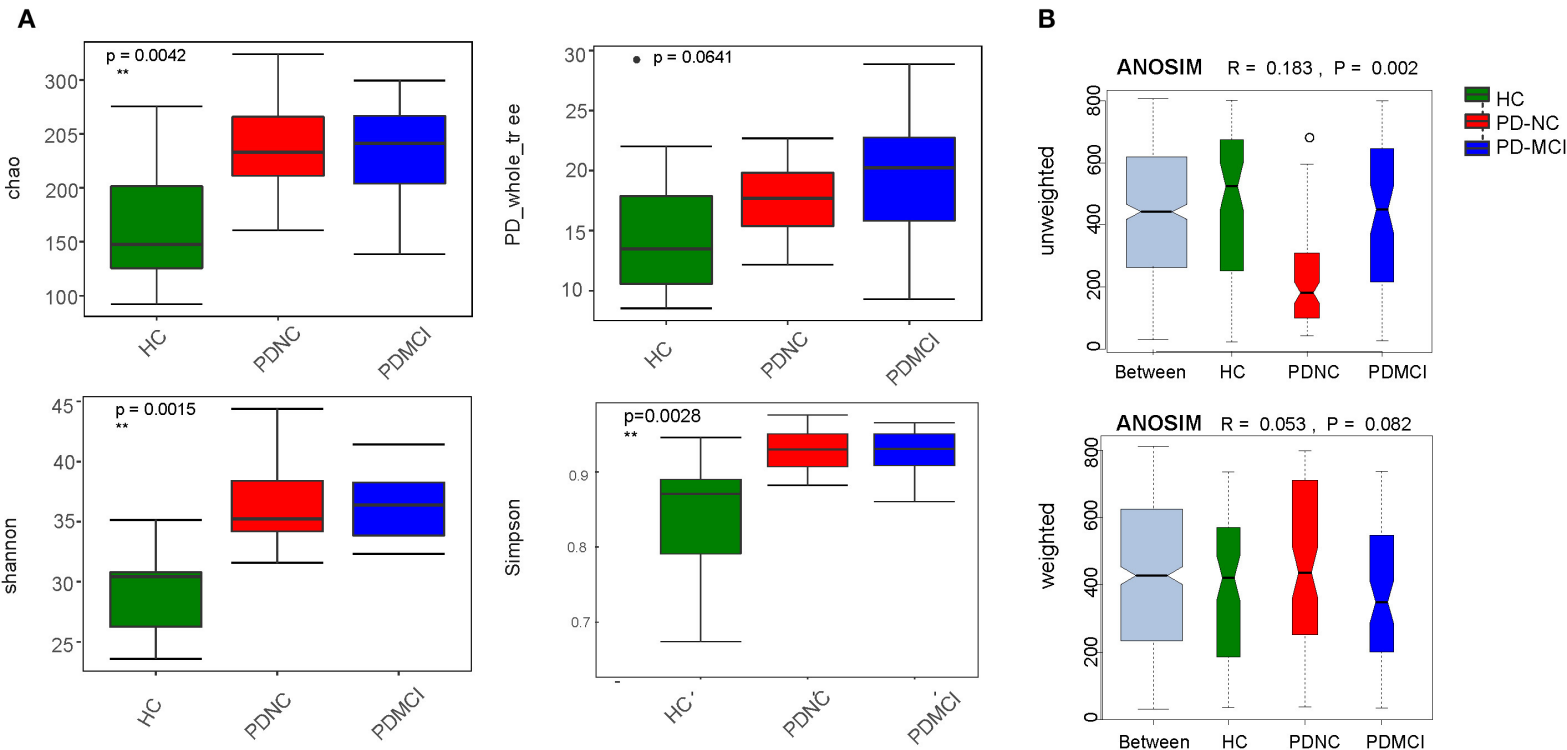
The alpha-diversity and beta-diversity indices of the fecal microbiome in the PD-MCI, PD-NC, and health group. **(A)** Box plots depict differences in the fecal microbiome diversity indices among three groups according to the Chao 1 index, PD whole tree index, Shannon index and Simpson index based on the OTU counts. Each box plot represents the median, interquartile range, minimum, and maximum values. **(B)** Unweighted and weighted ANOSIMs Unifrac analysis based on the distance matrix of UniFrac dissimilarity of the fecal microbial communities in the three groups. Respective ANOSIM R values show the community variation between three groups and significant P values are indicated. The axes represent the two dimensions explaining the greatest proportion proportion of variance in the communities. OTU, operational taxonomic unit, ANOSIM, analyses of similarities.

On average, about 33,259 (±3809; median 29,694) read pairs were sequenced per sample. In total, 561 different OTUs were identified across the 40 samples. The full dataset included bacteria from 108 genera, 42 families, 24 orders, 19 classes, and 11 phyla. The phylum Bacteroidetes was typically the dominant phylum in the gut microbiome (Figure S2). As for fecal microbiota, the mean community alpha-diversity indexes were significantly higher in the PD-NC and PD-MCI groups than in the HC group (Chao, Simpson, ACE, and Shannon indexes). Moreover, the PD_whole tree index was obviously higher in the PD-MCI group than in the HC group. However, no significant difference in alpha-diversity index was found between the PD-NC and PD-MCI groups. An obvious discrepancy was also found in beta-diversity based on the unweighted UniFrac ANOSIM metric (qualitative, ANOSIM *R* = 0.183, *P* = 0.002), but not the weighted UniFrac ANOSIM metric (quantitative, ANOSIM *R* = 0.053, *P* = 0.082) among the PD-MCI, PD-NC, and HC groups. Furthermore, based on the UniFrac index (PERMANOVA analysis on weighted UniFrac—HC vs. PD-NC: *R*^2^ = 0.118, *P* = 0.004; HC vs. PD-MCI: *R*^2^ = 0.086, *P* = 0.059; and PD-MCI vs. PD-NC: *R*^2^ = 0.037, *P* = 0.438; unweighted UniFrac—HC vs. PD-NC: *R*^2^ = 0.119, *P* = 0.001; HC vs. PD-MCI: *R*^2^ = 0.074, *P* = 0.065; and PD-MCI vs. PD-NC: *R*^2^ = 0.078, *P* = 0.039), the structures of fecal microbiota were found to be significantly different between PD-MCI and PD-NC groups (Figure 1).

### Alteration of Fecal Microbiota

The results suggested a remarkable difference in fecal microbiota among the PD-MCI, PD-NC, and healthy groups based on the LDA LEfSe analysis. The LDA analysis is often used to identify the presence and effect size of region-specific OTUs among different groups. After the LDA method, a higher relative abundance of the genus *Veillonella* and was detected in the HC group compared with the PD-NC and PD-MCI groups, whereas the abundance of the genera *Blautia* and *Ruminococcus* was higher in the PD-NC group compared with the remaining two groups. Additionally, the relative abundance of genera *Alistipes, Barnesiella, Butyricimonas, Odoribacter*, and *Anaerotruncus* was higher in the PD-MCI group compared with the other two groups (Figure S3).

### Generalized Linear Model

The GLM was used to model the microbiota that were significantly different at multiple taxonomic levels among the three groups after controlling for possible confounding factors (age, gender, BMI, education, and constipation). At the phylum level, the abundance of Bacteroidetes was obviously higher in the HC group than in the other two groups, while Actinobacteria was more abundant in the PD-MCI group. Particularly, the main differences between feces from the PD-MCI, PD-NC, and HC groups were associated with the genera *Alistipes, Barnesiella, Butyricimonas*, and *Odoribacter* (*P* < 0.05), suggesting that these microbiota were associated with PD-MCI. Additionally, the alteration in the abundance of genus *Blautia* (class Clostridia) and *Ruminococcus* obviously increased in the PD-NC group compared with the PD-MCI group (*P* < 0.05) (Table 2).

**Table 2:** GLMs for fecal at multiple taxons based on differences between the PD-MCI, PD-NC, and healthy groups.

**Group**	**Names**	**HC**		**PD-NC**		**PD-MCI**		***b*-value**	**95% CI**	***P-*value**
		**Mean**	**SD**	**Mean**	**SD**	**Mean**	**SD**			
HC	p__Bacteroidetes	0.564046999	0.113589923	0.402128905	0.147438829	0.48026331	0.121810186	0	−998.033260567641 to 998.033260567641	1.137e-06
	o__Bacteroidales	0.564042053	0.113597935	0.401756967	0.147520749	0.47924935	0.122609195	0	−998.033260567641 to 998.033260567641	1.137e-06
PD-MCI	f__Rikenellaceae	0.006425124	0.011347647	0.020687389	0.026993264	0.02967223	0.026466718	−39.8473849333268	−10941342.7946813 to 10941263.0999115	1.3025e-06
	g__Alistipes	0.006425124	0.011347647	0.020687389	0.026993264	0.02967223	0.026466718	−39.8473849333268	−10941342.7946813 to 10941263.0999115	1.3025e-06
	g__Odoribacter	0.000900909	0.001422584	0.002948696	0.002723746	0.00412749	0.003971608	−52.6246355204889	−1050.65789608813 to 945.408625047152	1.137e-06
	g__Barnesiella	3.0488E-05	0.000102306	0.004597766	0.00878484	0.0105767	0.020618392	−431.814685623343	−32925434976288792 to 32925434976287928	8.0939e-06
	g__Butyricimonas	0.001504989	0.002013678	0.007188302	0.008751651	0.00760577	0.006425853	−28.0999953907513	−302470.142449766 to 302413.942458985	1.2346e-06
PD-NC	p__Actinobacteria	0.004058595	0.004839057	0.015536206	0.018817958	0.01553621	0.018817958	18.0978142678544	−979.935446299786 to 1016.1310748355	1.137e-06
	c__Clostridia	0.209882136	0.15364877	0.362103725	0.153287927	0.32155621	0.1598763	0	−32925434976288360 to 32925434976288360	8.0939e-06
	o__Clostridiales	0.209882136	0.15364877	0.362103725	0.153287927	0.32155621	0.1598763	0	−998.033260567641 to 998.033260567641	1.137e-06
	f__Ruminococcaceae	0.087099771	0.079416241	0.214225472	0.112880227	0.19383011	0.141379614	1.06927265837216e-22	−998.033260567641 to 998.033260567641	1.137e-06
	g__Ruminococcus	0.013865641	0.029772867	0.042217669	0.051187442	0.02865663	0.031597992	−212.234360350757	−229.783877868329 to −194.684842833186	1.1406e-06
	g__Blautia	0.012762124	0.010803305	0.026715962	0.021761436	0.00752145	0.012040004	−2.99712948530676	−394854347.322569 to 394854341.32831	1.3046e-06

Result of the GLMs for significant phylum, class, order, family and genera (sequence counts) based on the group factors and possible confounding factors (age, gender, BMI education and constipation) of 40 individuals.

The b-value (positive number) indicated the taxa were associated with PD-MCI and PD-NC patients.

*GLM, general linear model; CI, confidence interval; BMI, body mass index*.

### Predictive Function Analysis

PICRUSt based on closed-reference OTU was used to predict the abundances of functional categories in the KEGG ortholog (KO). In this study, 664 KOs having significantly different abundances were identified between PD and HC fecal samples. A plot of top 20 KOs identified with significantly different abundances in the fecal microbiota among the three groups (FDR, *P* < 0.05) was made. Most reference pathways had more genes in HC compared with patients with PD, particularly pathways involved with energy metabolism, metabolism of cofactors and vitamins, glycan biosynthesis and metabolism, and metabolism of other amino acids in the level 2 KEGG pathway. The microbial gene functions related to membrane transport, including transporters, ATP-binding cassette (ABC) transporters, transcription factors, and benzoate degeneration, in the level 3 KEGG pathway were higher in the fecal microbiome of the PD-MCI group. Additionally, the microbial gene functions related to glycerophospholipid metabolism, base excision repair, and signal transduction mechanism in the level 3 KEGG pathway were higher in the fecal microbiome of the PD-NC group (Figures S4A–C).

### Association Between Altered Microbiota and Cognitive Ability

Mostly, the abundance of altered fecal microbiota showed a negative association with cognitive performance. The genera *Ruminococcus, Bilophila, Desulfovibrio, Barnesiella, Butyricimonas, Acidaminococcus, Pyramidobacter*, and *Oxalobacter* were negatively associated with the MMSE scores. In addition, the genera *Alistipes, Sutterella, Odoribacter, Butyricimonas, Hungatella, Helicobacter, Solobacterium, Oscillospira*, and *Hydrogenoanaerobacterium* were negatively associated with the MoCA scores (Figure S5). No significant difference in the SCFA level was found among the three groups (Table S1). However, the isovaleric and isobutyric levels negatively correlated with the MMSE scores (Figure S6).

## Discussion

The differences in gut microbiota composition among Chinese patients with PD-MCI and PD-NC were not explored by previous studies. This study was novel in showing that the composition of gut microbiota changed in the PD-MCI group compared with the PD-NC and HC groups in the Chinese population. Both alpha-diversity and beta-diversity indexes in this study provided powerful evidence that the gut microbiota in patients with PD were different from those in HC, which was consistent with the results of previous studies (6, 13, 17, 18). Additionally, although no statistically significant differences were found with respect to commonly used alpha-diversity indices in the PD-MCI and PD-NC groups, this study confirmed the significant differences in beta-diversity indexes, particularly at the genus level, between the PD-MCI and PD-NC groups. Taken together, this study provided powerful evidence that the gut microbiota in PD-MCI were different from those of PD-NC and HC.

Bajaj and coworkers claimed that the Porphyromonadaceae family was associated with a poor cognitive performance (19). Recently, an increased abundance of *Porphyromonas gingivalis* was found in the feces of patients with Alzheimer's disease (20). According to the report of Lee and Trojanowski (21), PD shared similar pathological changes with AD, such as neurofibrillary tangles, amyloid-beta plaques, and tau propagation, which might accelerate the process of cognitive decline in PD. Then, consistent with former studies on gut microbiota in patients with PD (7, 8, 13, 18), a significant higher abundance of several genera of the Porphyromonadaceae family, including *Barnesiella, Butyricimonas*, and *Odoribacter* as well as *Alistipes* from the Rikenellaceae family was found in the PD-MCI group after GLM analysis in this study. Furthermore, this study found that the genus *Butyricimonas* negatively correlated with the MMSE and MoCA scores, in line with the results of a previous study in China (13). Also, the genus *Barnesiella* negatively correlated with the MMSE scores. Further, genera *Alistipes* and *Odoribacter* negatively correlated with the MoCA scores.

Consistent with the recent studies reporting a decrease in the abundance of the Lachnospiraceae family and genus *Blautia* in the feces of patients with PD (6, 7), this study found a significantly lower abundance of genus *Blautia* in the feces of the PD-MCI group compared with that in the PD-NC group. Although the correlation between genus *Blautia* and cognition performance was not observed in this study, it was reported that decreased abundance of the Lachnospiraceae family was associated with cognitive decline (7, 14). Nevertheless, the abundance of genus *Blautia* in the feces was higher in the patients with PD-NC compared with that in HC. As genus *Blautia* is the main force of SCFA-producing bacteria, it may prevent PD and reverse the disease as a compensatory mechanism in patients with PD-NC who are in the earlier stage of PD. To conclude, a further study on patients with different PD duration is needed to clarify the presence of genus *Blautia*.

Moreover, the functional interpretation of the intestinal microbiome demonstrated that the progressive enrichment of the modules for membrane transport in patients with PD-MCI suggested a potentially active communication between the microbiota and the host. Membrane transport pathways, such as those involving transporters and ABC transporters, are essential to cell viability and growth (22). Moreover, the ABC efflux transporters have two contradictory effects on the development and progression of neurological diseases. On the one hand, they protect the central nervous system (CNS) by promoting detoxification, but also constitute an obstacle to brain penetration, diffusion, and bioavailability of CNS therapeutics (23). The enriched modules for membrane transport were also found in patients with AD (14). The ABC transporter A1 (ABCA1) provides transcriptional and translational evidence that the expression of ABCA1, a key modulator of cholesterol transport across the plasma membrane, is dysregulated in the brain of patients with AD and this dysregulation is associated with increased severity of AD, whether measured functionally as dementia severity or neuropathologically as increased neuritic plaque and neurofibrillary tangle density (24). Nevertheless, the association between cognition decline in patients with PD and membrane transporters should be further studied because the functional prediction analysis in this study was based on OUT in partial 16s RNA and not very reliable compared with metagenomics.

SCFAs were found to be the main factors inducing microglial activation and acceleration, indicating the role of acceleration of SCFA deficiency in the pathogenesis of PD (4, 25). SCFAs are made by bacteria in the gut, notably those belonging to the family Lachnospiraceae. Although no significant difference in SCFA concentration was found among the three groups in this study, genus *Blautia*, which belongs to the Lachnospiraceae family, was reported to be depleted in the PD-MCI group compared with the HC group. To some extent, a shortage of SCFA may be a common consequence of illness rather than a specific cause or even a biomarker of PD (6). This is because the depletion of SCFA and SCFA-producing organisms has been observed in diverse disorders. On the contrary, this study revealed alterations in at least six genera of bacteria and numerous metabolic pathways among the three groups, indicating that there was more to the microbiome dysbiosis in PD with cognition decline than SCFA discrepancy.

A strength of this study was the recruitment of healthy spouses as controls who shared the same direct environment and diet; also, the individuals enrolled were all Cantonese people with a balanced diet. Moreover, the influence of anti-Parkinson's medicines was taken into consideration in this study. According to Scheperjans and colleagues, Catechol-O-methyltransferase inhibitors were the only anti-parkinsonian drug significantly associated with the abundance of Enterobacteriaceae, which did not show a difference among the three groups in this study either (18). Elucidating the differences in the fecal microbiota composition of the patients with PD-MCI may improve the understanding of the pathogenesis of PD with cognitive impairment, provide a foundation to predict the development of PDD, and help find a novel treatment for cognition decline in patients with PD. Nevertheless, apart from the limited sample size, a bias might be caused by the higher prevalence of healthy spouses in women in this study. Therefore, additional gender-balanced large-scale studies on participants with cognitive impairment in different domains are needed to validate the findings of this study. The relationships between constipation, dietary habits, distinct microbiota in patients with PD, and SCFA concentrations were not detected in this study, which need further exploration.

## Data Availability Statement

The datasets generated for this study can be found in the National Center for Biotechnology Information (NCBI) BioProject database with project number PRJNA561023.

## Ethics Statement

The studies involving human participants were reviewed and approved by Guangdong Provincial People's Hospital. The patients/participants provided their written informed consent to participate in this study.

## Author Contributions

Conception: KN, LW, and TR. Organization: KN and TR. Execution: TR, JZ, QZ, and SJ. Statistical analysis and design: TR and YQ. Execution: TR and JC. Review and critique: YZ, YG, LW, and KN. Manuscript preparation and writing of the first draft: TR and SJ.

### Conflict of Interest

The authors declare that the research was conducted in the absence of any commercial or financial relationships that could be construed as a potential conflict of interest.
