# Supplementary material for: Gut Microbiota Altered in Mild Cognitive Impairment Compared With Normal Cognition in Sporadic Parkinson's Disease
Source: Front Neurol. 2020 Feb 25;11:137. doi: 10.3389/fneur.2020.00137 (PMC7052381; doi:10.3389/fneur.2020.00137)
Supplement: Supplementary file 1 [file Data_Sheet_1.zip › Figure S7.PDF]

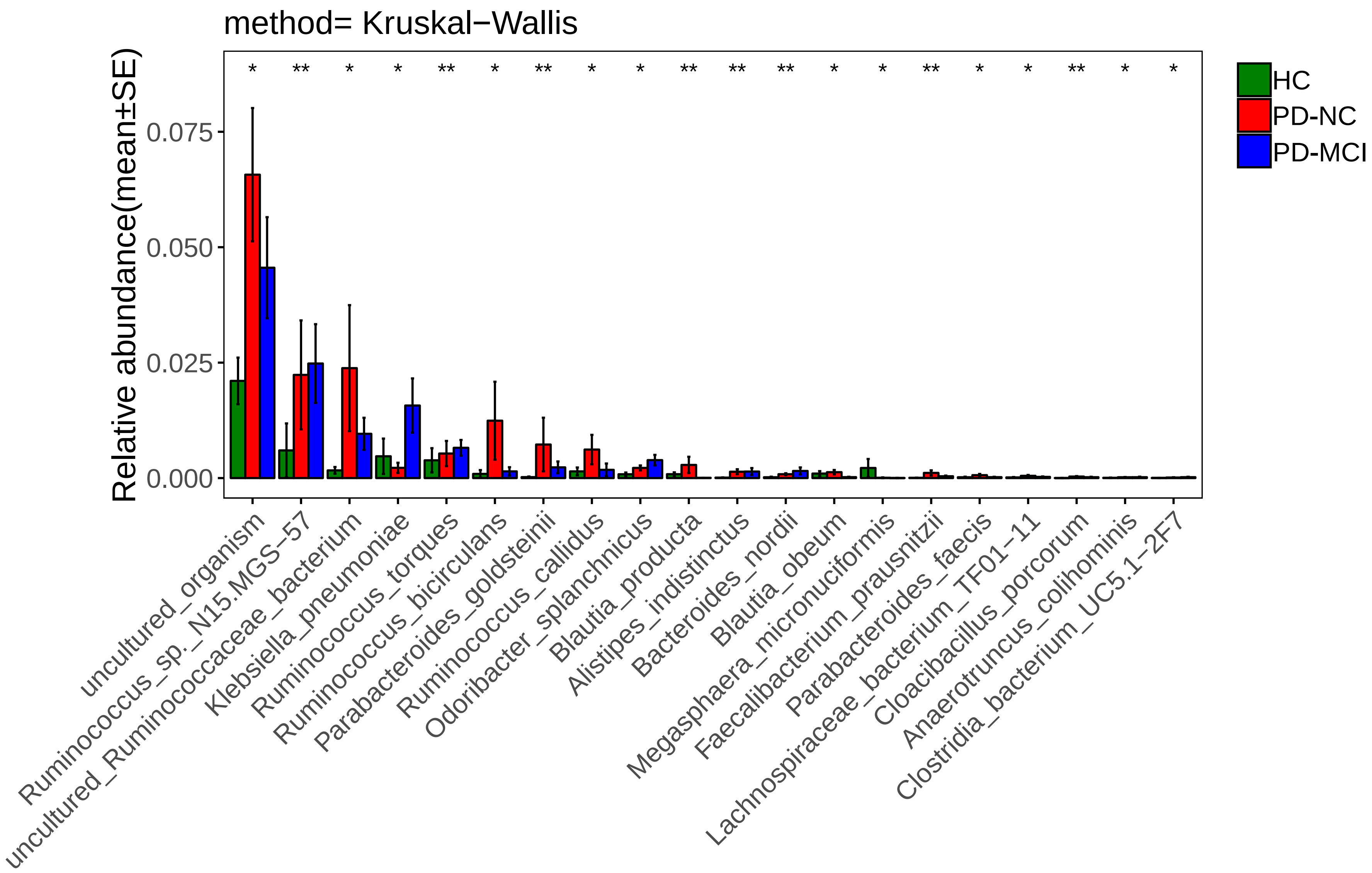

Figure S7: Significant microbiome (plot top 20) at spieces level after statistic method of Kruskal −Wallis. *P<0.05; **P<0.01.
