# Supplementary figures and images for: Gut Microbiota Altered in Mild Cognitive Impairment Compared With Normal Cognition in Sporadic Parkinson's Disease
Source: Front Neurol. 2020 Feb 25;11:137. doi: 10.3389/fneur.2020.00137 (PMC7052381; doi:10.3389/fneur.2020.00137)

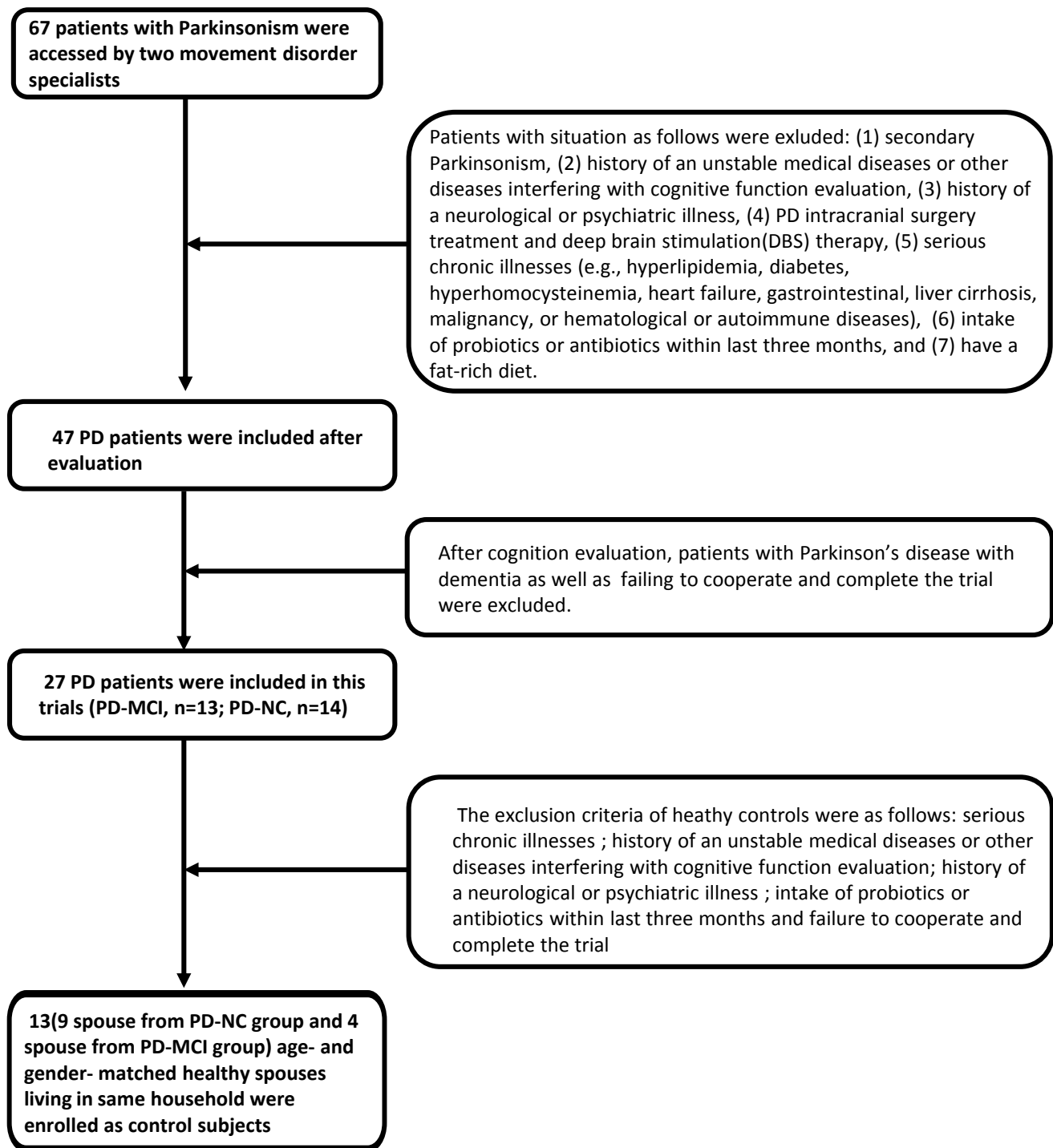

**Figure S1. Flow diagram of recruiting patients with PD-NC, PD-MCI and healthy controls.**

Supplement: Supplementary file 1 [file Data_Sheet_1.zip › Figure S1.PDF]

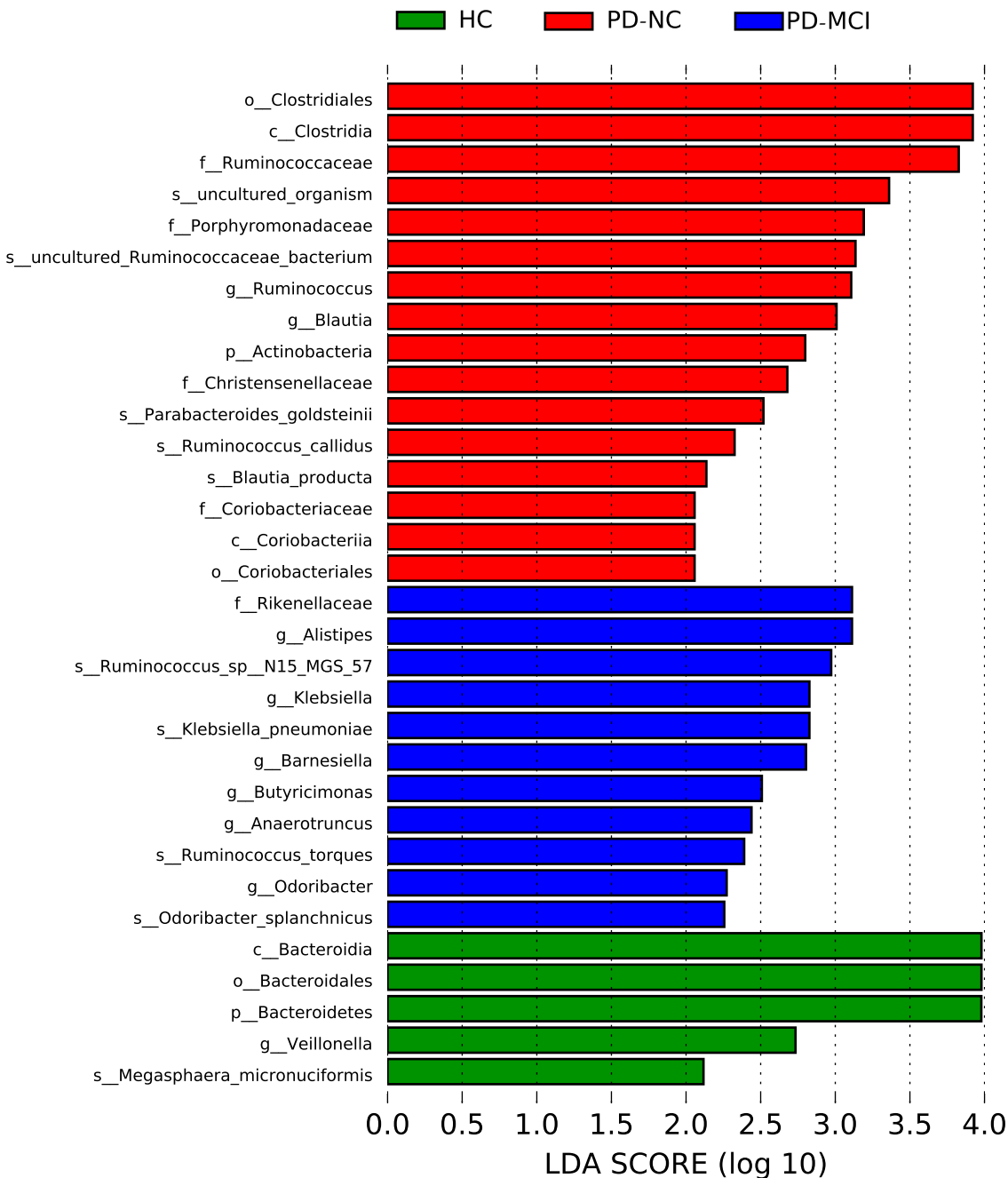

Figure S3. Alteration in the taxa between PD-MCI, PD-NC and healthy groups(LDA)

Supplement: Supplementary file 1 [file Data_Sheet_1.zip › Figure S3.PDF]

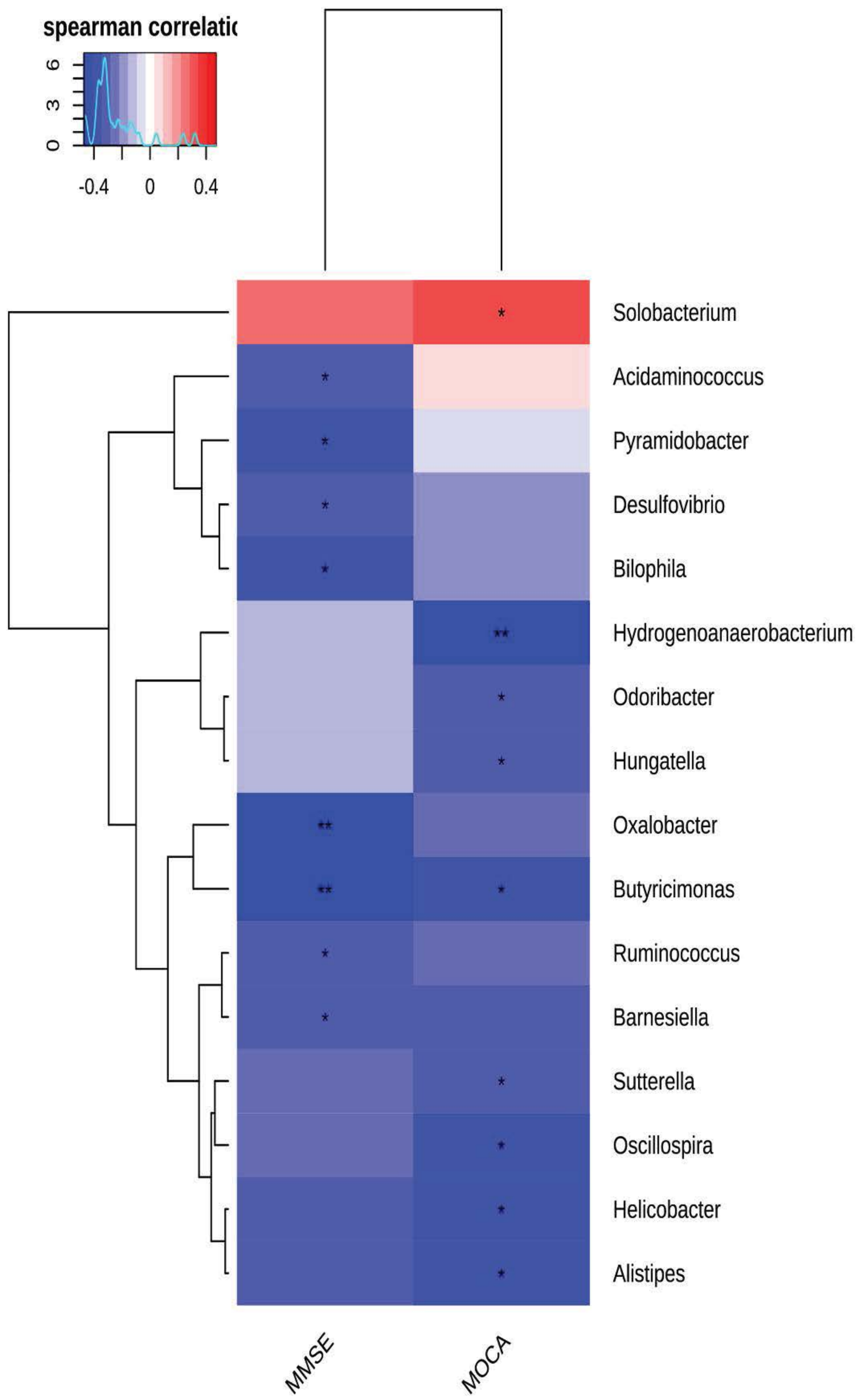

Figure S5. Correlation between fecal microbiota and MMSE, MoCA scores. \* $P < 0.05$ ; \*\* $P < 0.01$ .

Supplement: Supplementary file 1 [file Data_Sheet_1.zip › Figure S5.PDF]

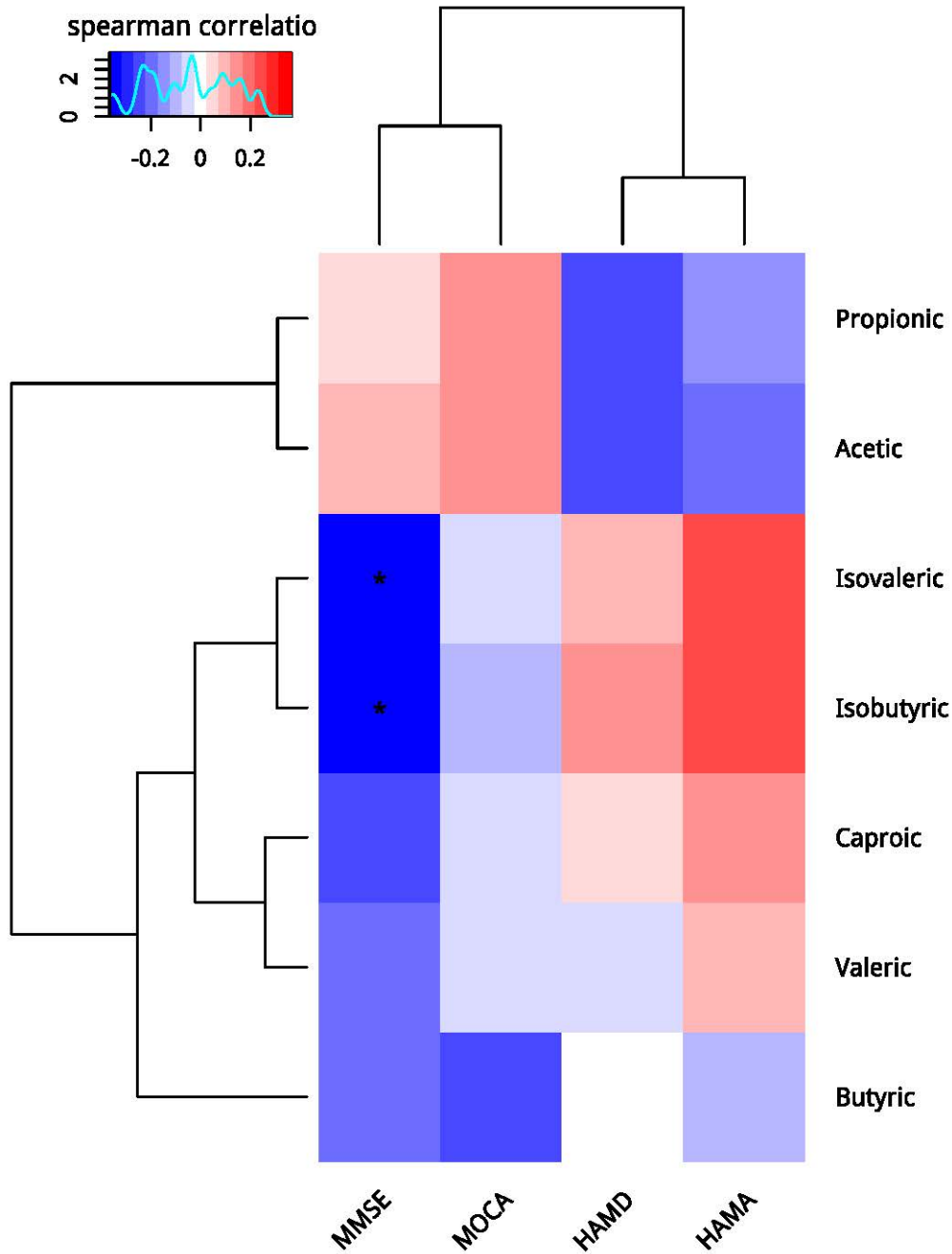

Figure S6. Association between SCFAs and cognitive ability. \* $P < 0.05$ .

Supplement: Supplementary file 1 [file Data_Sheet_1.zip › Figure S6.PDF]

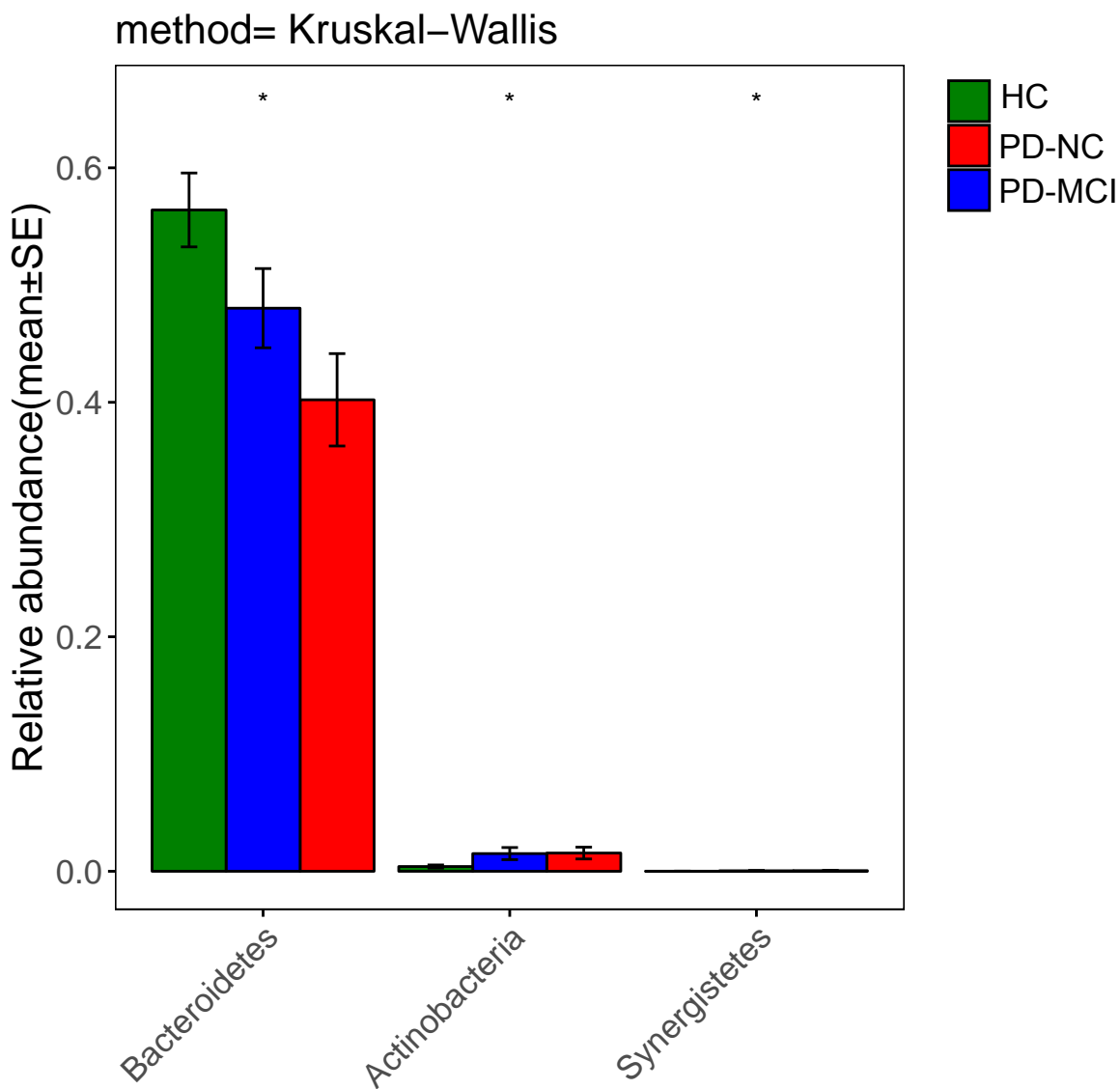

**Figure S2. Bacteroidetes were typically the dominant phyla in PD-NC, PD-MCI and healthy controls. \*P<0.05.**

Supplement: Supplementary file 1 [file Data_Sheet_1.zip › Figure S2.pdf]
